# Increasing participation of underrepresented groups in cancer early detection research: a scoping review

**DOI:** 10.3389/fpubh.2026.1841107

**Published:** 2026-08-19

**Authors:** Frederike Brockhoven, Maya Raphael, Nora Pashayan, Ignacia Arteaga

**Affiliations:** 1 Department of International Development, University of Oxford, Oxford, United Kingdom; 2 NIHR Bioresource, University of Cambridge, Cambridge, United Kingdom; 3 Department of Public Health and Primary Care, University of Cambridge, Cambridge, United Kingdom; 4 Institute for Health Policy Studies, University of California, San Francisco, San Francisco, CA, United States; 5 Early Cancer Institute, University of Cambridge, Cambridge, United Kingdom

**Keywords:** cancer research, diversity, early detection, minoritized groups, research culture, research participation

## Abstract

**Background:**

Improvements in access to early-detection research on cancer are still urgently needed to ensure that new research on early-stage cancer detection benefits all groups in society. To achieve this, cancer early detection (ED) studies must include participants from all walks of life. There are unique aspects to cancer early detection research that may deter potential research participants and complicate efforts to involve people from underrepresented backgrounds that require a review on its own merit. For instance, a unique risk for cancer ED research is overdiagnosis and overtreatment, in which a tumor is uncovered and treated that would not have led to the patient’s death if left undiscovered and untreated. This potential ‘side effect’ of cancer ED research participation is particularly problematic for those without adequate access to healthcare and insurance.

**Methods:**

We conducted a targeted scoping review to identify empirically tested approaches to improve participation of underrepresented groups in cancer early detection research. Searches were conducted in PubMed and PsycINFO using terms related to cancer, research participation, and underserved populations in the title and/or abstract. Eligible studies were peer-reviewed, published between 2002 and April 2022, conducted in high-income countries, focused on adults without a cancer diagnosis, and reported on their evaluation of an intervention designed to improve recruitment or participation of minoritized groups in cancer early detection research. Data were extracted on study characteristics, barriers to participation, intervention strategies, and outcomes of the recruitment and engagement intervention that was assessed. We analyzed data extracted using narrative synthesis to identify cross-cutting themes across barriers to participating, and recruitment or engagement approaches.

**Results:**

This review identified themes in the 38 included studies that aimed to recruit and involve participants from underserved groups in cancer ED research so that future studies may learn from or further test these varied strategies. We narratively grouped the review in terms of the barriers identified, and the approaches that have been designed to improve participation. These included rethinking recruitment locations and partnerships with local communities, designing educational interventions, combining research with community needs, increasing cultural competence of research teams, and overcoming practical barriers in study design.

**Conclusion:**

This scoping literature review highlights various tools, empirically tested, that research teams can employ to improve participation rates of groups underrepresented in cancer ED research. Combinations of these methods could help overcome the perceived barriers to participation in cancer research that mainly affect people without a cancer diagnosis from these minoritized groups. Not only would these methods increase the generalizability and representativeness of studies; the highlighted approaches also contribute to a more significant shift in research culture toward less extractive and more trusting relationships between researchers and the public.

## 1 Introduction

Cancer early detection and diagnosis are vital for improving the prospects of cancer patients. When cancer or pre-cancerous lesions are detected earlier, patients’ chances of surviving their disease are dramatically higher (1). Hence, cancer ED research has become a priority of cancer research in the United Kingdom (2), the United States (3), and other countries (4). Yet, improvements in participation to early-detection research on cancer are still urgently needed to ensure that new research on early-stage cancer detection benefits all groups in society (2). To achieve this, cancer ED studies must include participants from all walks of life. Similarly to many other health research fields, people from ethnic minorities and other underserved and minoritized groups are usually underrepresented in cancer ED studies (5–7).

There are unique aspects to cancer early detection research that may deter potential research participants and complicate efforts to involve people from underrepresented backgrounds (8, 9). Firstly, cancer ED research usually requires participants who are cancer free or asymptomatic, which thus requires ‘healthy’ people to consider the possibility of developing cancer. Having to think about a potentially lethal disease can put people off participation, especially those who already struggle to access adequate healthcare. This makes participation in cancer ED research benefit from a different recruitment process from other cancer clinical trials, which can recruit cancer patients already being treated in hospitals who are aware of their condition. Secondly, cancer-free and symptomatic people may be deterred by the invasive participation procedures that cancer ED research might require (10). Lastly, participating in cancer ED research can sometimes lead to the discovery of health problems, such as cancer or a higher risk of developing one. Overdiagnosis and overtreatment of slow-growing or indolent cancers; and detection of early cancers for which a treatment has not yet proven effective are known risks (11–14). Again, this potential ‘side effect’ of cancer ED research participation is particularly problematic for those without adequate access to healthcare and insurance (9).

These aspects of cancer ED research demonstrate the need for a scoping literature review about strategies to involve underrepresented groups in research in this particular field of cancer research. Although insights from systematic literature reviews and scoping reviews focusing on increasing participation of minoritized groups in cancer clinical trials (15–21) or clinical trials for other conditions (22–24) are beneficial, this scoping review seeks to map out only those tested interventions suitable for the involvement of underrepresented groups in the unique processes of cancer ED research.

This manuscript is part of a wider effort to develop recommendations for improving diversity and trust-building in cancer ED research. As part of ‘REPRESENT: A Community Engagement Roadmap to Improve Participant Representation in Cancer Research Early Detection’ (8, 9), we conducted a targeted scoping review to identify the approaches that have been implemented to improve participation of minoritized groups in cancer ED research.

## 2 Methods

This study reports on a targeted scoping review to map and analyze the wide range of interventions that have been tested, without excluding studies based on small sample sizes or heterogeneous evaluation methods (25). We developed narrative themes in the 38 included studies that aimed to recruit and involve participants from underserved groups in cancer ED research so that future studies may learn from or further evaluate these varied strategies.

### 2.1 Search strategy

The databases used for this systematic literature review, conducted in April 2022, were PubMed and PsycINFO. The question we sought to answer is: ‘What approaches have been implemented to increase participation of people without a cancer diagnosis from underrepresented groups in cancer early detection research?’. The search strategy consisted of four themes, each forming a set of related (MeSH) terms, shown below in Table 1: research (e.g., clinical trial), underrepresented communities (e.g., underserved, minorit*, African American, Black, Latin*), participation (e.g., engagement, involvement, recruit*), and cancer (e.g., oncol*, lymphoma). The category of ‘underrepresented communities’ included the highest number of terms to ensure a wide range of underserved groups were included in the search. These groups included various ethnic, indigenous, and linguistic minorities, gender and sexual minorities, people with physical disabilities and neurodiverse people, groups marginalized based on their geographic location or socio-economic status, and those often excluded based on their immigration status. Since there are also various terms to denote participation, each with a nuance that can differ between countries and research contexts, it was decided to include all terms that indicate some level of involvement in research. Search strategies and filters were modified for the corresponding search database.

**Table 1:** Search strategy via PubMED and PsycINFO using medical subject headings (MeSH) terms.

Medical Subject Headings (MESH) Terms used
(1) Research
(“clinical trial”[Title/Abstract]) OR (“early detection research”[Title/Abstract]) OR (“cancer research”[Title/Abstract])
(2) Underrepresented communities
(underrepresent*[Title/Abstract]) OR (minorit* [Title/Abstract]) OR (underserved[Title/Abstract]) OR (hard-to-reach[Title/Abstract]) OR (marginal*[Title/Abstract]) OR (vulnerable[Title/Abstract]) OR (disadvantaged[Title/Abstract]) OR (racial*[Title/Abstract]) OR (ethnic*[Title/Abstract]) OR (BIPOC[Title/Abstract]) OR (BAME[Title/Abstract]) OR (“South Asian”[Title/Abstract]) OR (Asian[Title/Abstract]) OR (“African American”[Title/Abstract]) OR (Latin*[Title/Abstract]) OR (Black[Title/Abstract]) OR (Hispanic[Title/Abstract]) OR (“South American”[Title/Abstract]) OR (Indigenous[Title/Abstract]) OR (“Native American”[Title/Abstract]) OR (Aboriginal[Title/Abstract]) OR (migrant[Title/Abstract]) OR (immigrant[Title/Abstract]) OR (disabil*[Title/Abstract]) OR (disabl*[Title/Abstract]) OR (neurodiverse[Title/Abstract]) OR (handicap*[Title/Abstract]) OR (sexual minorit*[Title/Abstract]) OR (gender minorit*[Title/Abstract]) OR (LGBT*[Title/Abstract]) OR (gay[Title/Abstract]) OR (lesbian[Title/Abstract]) OR (rural[Title/Abstract]) OR (“socio-economic status”[Title/Abstract]) OR (working-class[Title/Abstract])
(3) Participation
(recruit*[Title/Abstract]) OR (participa*[Title/Abstract]) OR (engagement[Title/Abstract]) OR (retention[Title/Abstract]) OR (involvement[Title/Abstract]) OR (enrolment[Title/Abstract]) OR (enrollment[Title/Abstract]) OR (accrual [Title/Abstract])
(4) Cancer
(cancer[Title/Abstract]) OR (oncol*[Title/Abstract]) OR (lymphoma[Title/Abstract]) OR (carcinoma[Title/Abstract]) OR (adenoma[Title/Abstract]) OR (melanoma[Title/Abstract]) OR (myeloma[Title/Abstract]) OR (sarcoma[Title/Abstract]) OR (leukemia[Title/Abstract]) OR (carcinoid[Title/Abstract]) OR (tumor[Title/Abstract]) OR (astrocytoma[Title/Abstract])

### 2.2 Inclusion and exclusion criteria

Inclusion criteria for the review were the following: (1) only published empirical peer-reviewed studies; (2) studies published between 2002 and April 2022; (3) research undertaken in a High-Income Country; (4) studies involving adult research participants (18 years or above); (5) studies directly related to cancer early detection research; (6) studies involving participants who have *not* yet been diagnosed with cancer; (7) research that is not primarily therapy-focused; (8) articles aiming to enhance the recruitment and participation of underserved groups; (9) studies that implement and intervention to improve recruitment or participation in cancer studies *and* quantitatively/qualitatively evaluate its effectiveness; and (10) articles published in English. The full list of inclusion and exclusion criteria used by the two reviewers is included on Table 2, and the instructions designed by one of the senior authors (NP) and followed by the authors screening the articles is attached as a supporting file.

**Table 2:** Inclusion and exclusion criteria.

Inclusion criteria	Exclusion criteria
Research undertaken in High-Income Country (e.g., USA, UK, Western Europe, Canada, Australia, New Zealand, Hong Kong)	Research undertaken in Low- or Middle-Income country
Participants aged 18 years or above	Participants under 18 years old
Published in or after 2002	Published before 2002
Peer reviewed publication	Not peer reviewed and/or not yet published
Paper relates directly to cancer—all cancers, gene mutations associated with cancer (e.g., *BRCA1*/*BRCA2*), viruses associated with cancer (e.g., HPV)	Paper does not relate to cancer
Purpose of study is not therapeutic/service but cancer research	Research is therapeutic—participants participate to better their own health outcomes
Research involves participants that have not been diagnosed with cancer (pre-diagnostic)	Research participants are diagnosed cancer patients or cancer survivors
Paper focuses on recruitment, participation, or engagement of underserved groups	Paper focuses on recruitment, participation or engagement of general public, not specific underserved groups
Intervention to improve recruitment, participation or engagement is implemented/tested	Intervention to improve recruitment, participation, or engagement is only suggested, not implemented/tested
Implemented intervention is evaluated, quantitatively or qualitatively	Implemented intervention is only described, outcomes or implementation is not quantitatively or qualitatively evaluated (e.g., protocol)

A few remarks to justify this approach. Criterion (6) was needed to keep the focus of the analysis on early detection research, narrowing the focus on those individuals who are cancer free and have not being diagnosed with cancer in the past, since those groups may have different incentives to participate in research and may have preexisting relationships with clinical teams running the studies. The review team realized that criterion ‘research that is not primarily therapy-focused’ was difficult to define, but preserving some homogeneity in the intent of the study was key to achieve the goals of the review. This criterion was included because studies centered on therapeutic interventions often involve distinct participation incentives, expectations, and engagement processes, which may not be comparable to those examined in non-therapy-focused research contexts. So, for example, if the research in question produced secondary incentives for participants (e.g., receiving results of mammogram), that manuscript was included, but if the purpose of the study was therapeutic (e.g., cancer patients have the chance to receive novel treatments), the manuscript was excluded. Furthermore, criterion (9) was essential to map effective engagement and recruitment strategies to improve participation of minoritized groups: if those strategies were named on the manuscripts but not assessed in quantitative or qualitative form.

**Figure 1:**
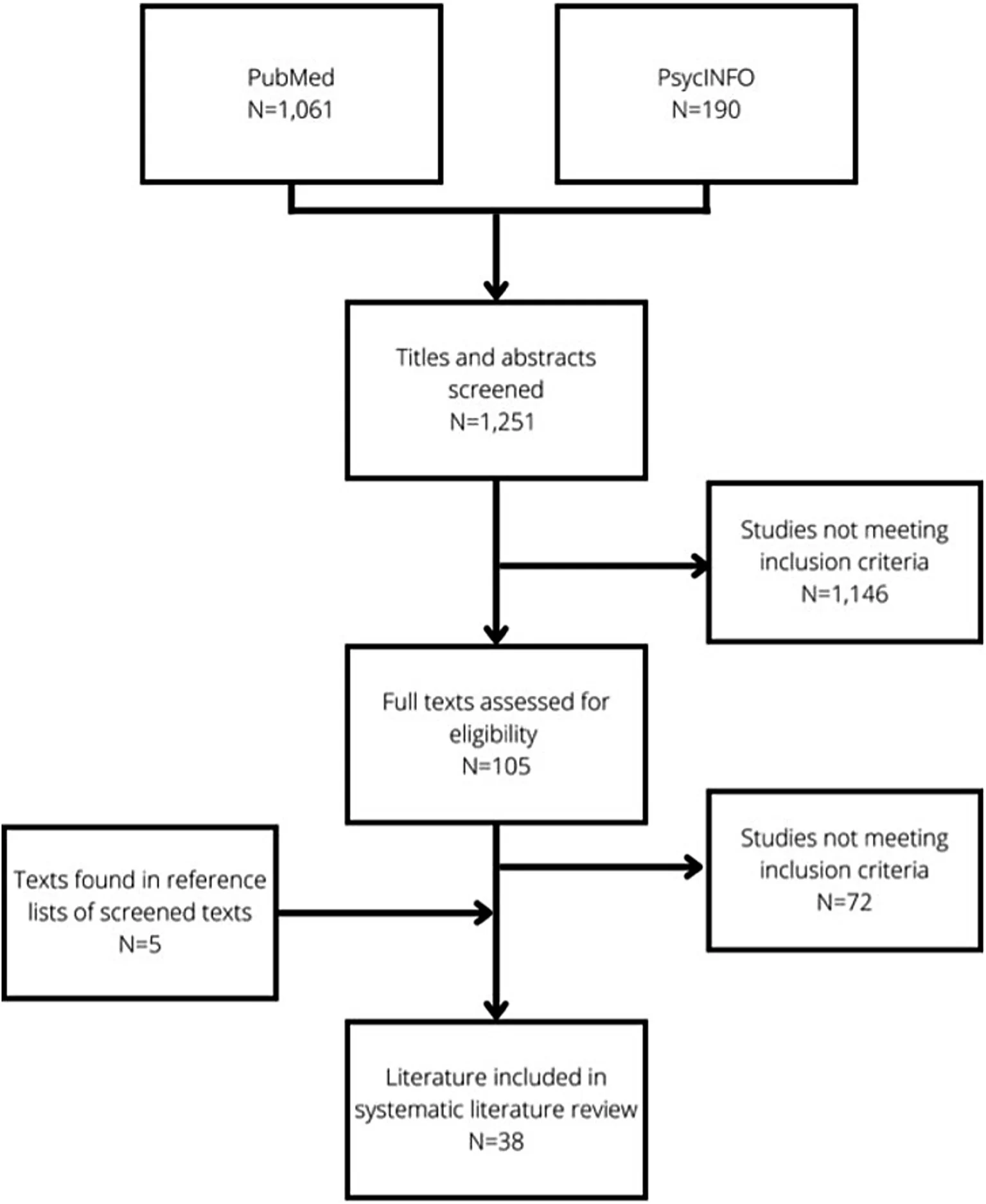
Flow chart of the review inclusion/exclusion process.

The search yielded 1,061 results on PubMed and 190 results on PsycINFO, whose titles were all scrutinized to filter out irrelevant papers. The abstracts of all remaining studies were read to select those that may fit the inclusion criteria. Figure 1 shows a flow chart detailing manuscript inclusion. Two investigators determined manuscripts’ eligibility independently: the first author (FB) screened and read all identified papers, and another (MR) independently screened a 25% sample of the 1,251 identified studies. Given that the agreement on inclusion and exclusion was satisfactory, the first author screened the rest of the papers alone. This process generated a sample of 33 articles, to which five more articles found in references of screened documents were added. This led to a final sample of 38 manuscripts. Figure 1 presents the flow chart describing the selection process.

### 2.3 Analysis

An iterative process was used to create a standardized framework for data extraction, followed by a narrative synthesis of the information presented in each manuscript. Information recorded for each study included basic study characteristics, including authors, publication year, target population, geographic location, cancer site, reported intervention or study, recruitment practices used, sample size, method to evaluate recruitment success, recruitment outcomes reported and funding source.

The narrative synthesis of the reported studies involved three steps: (1) extraction of barriers to participation, as well as intervention components that were originally assessed and reported on by each study. This included the outcomes of the assessment (i.e., whether the intervention was successful in increasing participation or enrollment of the targeted population); (2) inductive coding and grouping of conceptually related codes; and (3) development of higher-order themes that captured recurring approaches across studies. These themes are presented in the results section.

## 3 Results

### 3.1 Characteristics of included articles

Table 3 presents the summary of the dataset containing 38 studies published between 2002 and 2022, the Supplementary file includes the full dataset, including the studies’ recruitment approaches, identified barriers to participation, minoritized groups targeted, cancer site (if specified), evaluation method of the recruitment approach, and funding source. As shown on Table 2, the final selection of studies was overwhelmingly carried out in the United States, except for one study in the UK (26).

**Table 3:** Summary of characteristics of the studies included (*n* = 38).

**Citation**	**Study type**	**Cancer site (if specified)**	**Study/ Intervention reported**	**Participant recruitment practices**
Hainsworth, E. et al. (26)	Mixed methods	Prostate	Discussion panel of men with African/Caribbean ancestry to inform the development of a video that raises awareness and promotes research participation; culturally tailored video mailed to potential research participants and promoted online	Discussion panel of men with African/Caribbean ancestry to develop a video that raises awareness and promote research participation; culturally tailored video mailed to potential research participants and spread online
Webb Hooper, M. et al. (53)	Observational study	Unspecified	Community-engaged research projects initiated by the Case Comprehensive Cancer Center's community advisory board (CAB) that focused on concerns of importance to the community.	Community-engaged trust building project; community listening tour; participant-driven discussions with researchers about research, healthcare system, etc. At cancer centre
Cunningham-Erves, J. et al. (32)	Community-engaged pilot study	Unspecified	Educational intervention with video testimonials with minority community members and researchers at 13 town halls; training six community health educators to give educational interventions and eventually lead town halls	Educational intervention with video testimonials with minority community members and researchers at 13 town halls (7 Black and 6 Latino); recruitment for town halls flyers, newsletters and word-of-mouth; education about cancer prevention, research, why research is important for prevention and control of cancer; information about risks, benefits, successes and patient protection in research; training community health educators to give educational interventions and eventually lead town halls
Rogers et al. (46)	Exploratory case study	Colorectal	Qualitative screening-promotion study using 11 focus groups to explore the social determinants of screening uptake among Black men.	Community involvement; culturally tailored marketing materials; collaborating with community organisations; incentives ($20 gift card); recruitment at barbershops; partnership with barbershops and churches; recruitment via churches (flyers and from pulpit); (timing) event directly after Sunday service; providing food as incentive
Giri et al. (57)	Pilot intervention:	Unspecified	Group-based educational intervention at a community-based cultural centre in Philadelphia, using culturally tailored messaging and culturally-matched presenter.	Culturally tailored educational interventions; recruitment at community-based cultural centre in Philadelphia
Tabriz et al. (50)	Embedded trial	Colorectal	The e-assist: colon health trial conducted within an integrated health care delivery system. It used the health system’s EHR data and patient portal infrastructure to identify, recruit, and follow eligible patients.	EHR online patient portal; message from primary care physician to prompt screening
McElfish P.A. et al. (54)	Cross-sectional study	Breast	Mobile mammography van recruitment; community event recruitment; study recruitment during mammovan screening	Mobile mammography van recruitment; community event recruitment; recruitment during screening
Tan et al. (61)	Intervention	Unspecified	Culturally tailored educational intervention using live sessions, videos and brochures; multimodal community outreach through community partners.	Multimodal intervention; recruiters have relationship with community; face-to-face recruitment at church, community events, social clubs; recruitment via phone calls, text messages, emails; educational sessions at churches, universities, community centres; creation of culturally tailored video about biospecimen research; culturally tailored brochures;
McIntyre, J. et al. (56)	Randoized controlled trial	Unspecified	Randomized controlled trial to examine multiple communication channels for delivering cancer prevention and biobanking education to Hispanics	Eduducational intervention; education about biobanking and cancer prevention; materials in Spanish; educational video in Spanish; face-to-face discussion group for education; asking to sign up for biobanking after intervention
Davis et al. (33)	Observational study	Colorectal	Culturally-targeted, minimal intensity educational intervention on CRC screening using the fecal immunochemical test (FIT) among Blacks in community settings.	Recruitment through invited presentations at churches and community centres; recruitment at health fairs; approaching people at library, barber shop, public buildings, laundromats; door-to-door canvassing in Black neighbourhoods; culturally tailored flyers/brochures; radio and TV spots; advertising on Twitter, Facebook and Craigslist; ads in newspaper and magazines that serve Black community; direct mailing; snowball recruitment
Cudjoe, J. et al. (31)	Community based participatory reseach	Cervical	Community-based participatory genetic research study to educate, recruit, and retain African American women participants in genetic research	Recruitment in churches; recruitment through word of mouth; increasing cultural competence of research team; gender concordance; promoting altruism through health education; online recruitment via Whatsapp
Slade et al. (48)	Intervention/mixed methods	Prostate; breast; colorectal	Church leaders performed the functions of engaging, recruiting, and retaining peer educators. Church leaders were also asked to help their appointed Community Health Advisors engage, recruit, and retain qualified church members to participate in the educational workshops that were presented at their church site	Peer educators from church; community engagement; workshops organised by church to promote cancer early detection interventions; trust building
Smith, A. et al. (49)	Intervention	Breast	Community-based participatory genetic research study to enhance participation and breast cancer equity among African American women	Community-based participatory research; educational workshops; Q&A sessions; culturally appropriate marketing; events at diverse locations; recruitment at community events (e.G. 5K walk for breast cancer); development of partnerships with prominent breast cancer advocates
McNeill et al. (44)	Mixed-methods community-based participatory research	Unspecified	Community-based participatory research through which a partnership between a medical center and a set of churches was established. Partnership aimed at recruitment from churches; events to promote cancer (research) awareness; frequent results dissemination in ways that interest community; implementation of cancer control programmes and services; provide research experience to minority trainees interested in cancer disparities;	Community-based participatory research; recruitment and participation at churches; events to promote cancer (research) awareness; frequent results dissemination in ways that interest community; implementation of cancer control programmes and services; provide research experience to minority trainees interested in cancer disparities; organising fairs with church to recruit participants; pastors speak about research from pulpit;
Yeary, K.H.K., et al. (10)	Non-randomized controlled trial	Breast	A range of community outreach programs implemented in community spaces, including educational presentations about cancer and prevention, testimonies from community champions, presentation of research opportunities, and the opportunity to participate in research activities on-site	Community-based participatory approach; community-relevant event; women volunteered to take tests at different stations for their own benefit and then were asked to donate the results like blood sample, survey; intervention at church or community-based organisation; gift card for participation; education about metabolic syndrome and lifestyle fators to prevent cancer;
Tham et al. (51)	Pre-post assessment	Unspecified	Series of biospecimen education seminars for health care professionals and representatives from community organizations	Educational intervention to increase knowledge about biospecimen donation and its importance, half of participants work in healthcare
Dean, C. Et al. (62)	Mixed methods	Unspecified	Building trust with communities via mayor; endorsement of village council; local focus groups to design research; local 'champions' to help in recruitment; posts on Facebook (groups); flexible recruitment times	Building trust with communities via mayor; endorsement of village council; local focus groups to design research; local 'champions' to help in recruitment; posts on Facebook (groups); flexible recruitment times
Rodriguez et al. (45)	Descriptive observational study	Unspecified	Educational presentations on genomic research and the need for representation of diverse populations, followed by immediate opportunity to donate blood sample	Educational intervention on genomic research and the need for representation of diverse populations; educational intervention followed by immediate opportunity to donate blood sample; open events like health fair with direct opportunity to donate blood sample
Wallington et al. (52)	Mixed methods	Breast; HPV	Application of cultural competency frameworks to adapt clinical trial research in order to increase minority participation in nontherapeutic cancer clinical trials	Diverse staff; culturally competent study procedures; community access via gatekeepers; keeping research location close to where people work/live/study; creating community partnerships
Cupertino et al. (29)	Prospective cohort study	Unspecified	Capacity-building to support cancer health disparities studies among rural Latino communities: formation of a community advisory board (CAB), development of a cancer research training program for Spanish-speaking promotores from rural communities, and recruitment and training of a core of Spanish-speaking promotores to disseminate cancer research education throughout rural communities.	Community-based education; training promotores de salud to disseminate knowledge about cancer research in community
Jackson et al. (40)	Mixed methods	Prostate	In-person pilot education intervention on prostate cancer over several sessions with unaffected Black community members	Prostate cancer education programme; education about CCTs
Langford, A.T., et al. (43)	Intervention	Prostate; colorectal; lung;	In-person interactive educational intervention ("Men’s Fellowship Breakfast") to provide Black men evidence-based, timely health information about cancer risk reduction and health promotion behaviors, as well as other topics that attendees found important.	Breakfasts Saturday morning to disseminate information about trials, lifestyle, prevention and screening and other topics that men would find interesting; recruitment via Church, hospitals, social services agencies and universities; recruitment via paid newspaper and radio advertisements
Wang et al. (58)	Intervention	Breast; colorectal	Study reports on two studies, one based on recruitment in community settings (Promoting Adherence to Mammography Use in Chinese Women), and one based on clinical settings (Physician-based Trial to Increase Colorectal Cancer (CRC) Screening in Chinese Men and Women)	Bilingual staff; culturally and linguistically tailored materials; recruitment by Chinese American physicians; recruitment via letter and follow-up phone call; in-person recruitment at community events;
Ma et al. (59)	Descriptive observational study	Unspecified	Culturally and linguistically appropriate community-based educational intervention to increase knowledge of and intent to participate in cancer clinical trials among underrepresented Chinese Americans	Culturally and linguistically appropriate community-based educational intervention; recruitment of community representatives to become community health educators who organise education; workshops focus on benefits to science and to larger Asian American community
Greiner et al. (37)	Community based participatory reseach across a portfolio of clinical studies	Colorectal; breast; prostate;	Community-based participatory research in which communitiy members are involved in every stage of the intervention and promote the development of professional skills for those involved.	Recruitment in waiting rooms of primary health centres; low-literacy brochures; training community health promoters/promotores de salud; recruitment via trained community health promoters/promotores de salud; call at different times to reach participants/flexibility; social marketing campaign on Facebook/Twitter; recruiters from community; recruitment in churches, hair salons, health fairs, support groups, public library, mammography clinic, and a Black sorority; offering programme that benefits participants in research; word of mouth/snowball sampling; informal dissemination at barber shops, churches; promotores de salud deliver one-on-one education workshops; radio campaign; offering skill-building for participants
Kiviniemi (42)	Pilot intervention	Unspecified	Two strategies reported: a group educational intervention to increase knowledge about cancer research and the role of biobanking, and tabling events at community health fairs to encourage research participation in biobanking research projects.	Recruitment at health fair, recruitment after educational session; both via local cancer centre; biospecimens collected immediately after educational intervention
Colon-Otero, G. Et al. (30)	Prospective cohort study	Myeloma; breast	Twelve educational programs focused on multiple myeloma and five behaviors that reduce cancer risk, followed by the opportunity to fill out health surveys and donate blood samples at a mobile research unit.	Educational programme on healthy behaviour and cancer research in churches; samples taken for donator's own benefit but with option to donate for research
Friedman, D.B. (36)	Pilot intervention	Prostate	Educational intervention with black men and womenmto address the discordance between high rates of prostate cancer mortality and limited participation in cencer research	Community-based research design; educational intervention; radio recruitment; patient navigators; word of mouth recruitment; culturally appropriate language; flexible timing to work around participants' schedules
Leach et al. (60)	Descriptive observational study	Breast; cervical	Two case studies: a doctoral dissertation project on older women from rural Appalachia's knowledge and beliefs about breast cancer and their attitudes towards cancer screening; Faith Moves Mountains is a community-based participatory intervention designed to reduce the disproportionate burden of cervical cancer among Appalachian women by increasing Pap test use	Recruitment via churches; researchers attend community events; building trust over time; establishing a presence in community; recruitment via local senior citizen center; face-to-face recruitment; snowball recruitment; door-to-door canvassing; participating in volunteering efforts;
Jones, R.A. et al. (41)	Qualitative study	Prostate	Qualitative study was to explore the experiences of rural African American men in decision-making regarding prostate cancer screening	Recruitment at places where Black men are found: barbershops, community health centres, church; word-of-mouth recruitment; trusting relationships with community gatekeepers
Gren, L. Et al. (38)	Randomized controlled trial	Prostate; lung; colorectal; ovarian	Prostate, Lung, Colorectal and Ovarian Cancer Screening Trial: randomized controlled trial designed to evaluate whether certain screening tests reduce mortality from prostate, lung, colorectal, and ovarian cancer	Direct mail invitation to healthy people found in commercial mailing lists, department of motor vehicles, health plan members, voters' registrstion, AARP; community outreach via health fairs, churches, utility bills, union newsletters, 'invite a friend' flyers or referrals, enrollment seminars; ads in newspapers, on TV and on radio; word-of-mouth
Larkey et al. (55)	Randomized ommunity controlled trial	Cervical; breast; colorectal	Juntos en la Salud (Together in Health): a randomized, controlled community trial sought to test the effects on participants' behavior of two different, theory-based methods of implementing a promotora-led cancer prevention/screening curriculum.	Community-based; promotoras da salud/community health educators; culturally tailored education; community-based selection of recruitment sites; NO financial compensation
Fair et al. (34)	Retrospective case-control study	Breast	The Return after Mammography Study: hospital-based retrospective case control study using telephone interviews to examine factors that affect minority and medically underserved women’s follow up of incomplete mammography testing	More rigourous searches to find participants' contact details and attempt recruitment; call back multiple times until potential participant is reached
Sadler et al. (47)	Cross-sectional study	Breast	The Black Cosmetologists Promoting Health Program: A randomized controlled education trial	Conducting surveys at Black beauty salons; cosmetologist trained as health educator;
Kreling et al. (28)	Mixed methods	Breast; colorectal; cervical	Latin American Cancer Research Coalition: a model for cancer control focused on community-based clinics as the focal point for in-reach and community outreach targeted to Latinos to reduce cancer disparities	Multimodal intervention; increase knowledge about early detection and CCTs; develop media messages about need for screening; trust building in community clinics; bilingual and bicultural teams recruit in churches, stores, hair salons; health fairs and partner clinics
Halbert et al. (39)	Cross-sectional study	BRCA1 BRCA2 mutations, breast; cervical	Enrollment on cancer genetic counseling research based on clinic referral networks from community onlcology resources and oncology clinics	Recruitment in cancer centres; recruitment via community oncology resources; recruitment via health fairs
Sheppard et al. (27)	Retrospective analysis	Colorectal; breast; lung;	Latin American Cancer Research Coalition: a model for cancer control focused on community-based clinics as the focal point for in-reach and community outreach targeted to Latinos to reduce cancer disparities	Latino researchers and providers; culturally tailored materials released through culturally appropriate outlets such as Latino radio stations.
Ford et al. (35)	Randomized trial	Prostate; lung; colorectal	A randomized trial of recruitment methods for older African American men in the Prostate, Lung, Colorectal and Ovarian Cancer Screening Trial	Personal contact; team members of similar ethnicity; churches as study sites; transportation to churches

Most studies were funded, in part or in full, by the National Institutes of Health, demonstrating the emphasis that National Cancer Institute, as well as other national institutes, historically had on promoting diversity in recruitment during at least part of the review period. For example, several studies report on outcomes of awards funded by a U54 federal grant mechanism specifically used to improve health disparities affecting minoritized groups by implementing community-based research approaches, such as the ongoing collaboration with community advisory boards and the development of long term community partnerships (27–29).

In addition, studies often focused on similar minoritized groups: African American (10, 26, 30–53) and Latinx or Hispanics (28, 29, 32, 37, 38, 45, 50, 51, 54–56). Studies focusing on Asian and Indigenous groups were reported on at a lesser extent (57–60). However, category assignment was not exclusive, as some studies usually included multiple underrepresented groups simultaneously in their intervention.

Those studies that sought participation in research on specific cancer types predominantly focused on breast (10, 27, 28, 30, 34, 47–49, 52, 54, 55, 58, 60), colorectal (27, 33, 35, 37, 43, 46, 50, 58), prostate (26, 35, 36, 38, 40, 41, 43, 48) and lung (35, 38, 43) cancers, sometimes in combination. However, no cancer type was specified in many cases, This was the case of studies focusing on enhancing diversity in biospecimen collection (42, 45, 51, 61).

The methodological approaches used to assess the effectiveness of recruitment approaches across the 38 included studies were predominantly quantitative (*n* = 19, 50%) or mixed methods (*n* = 17, 45%), with only two studies relying primarily on qualitative methods. Quantitative outcomes most commonly assessed were rates of recruitment, enrollment and participation; or change in knowledge, attitudes, or willingness to engage in biospecimen donation, while mixed-methods studies frequently combined these measures with qualitative data from focus groups, interviews, community discussions, or participant feedback. Community-based participatory research approaches were common across the literature. Reported study designs included randomized and non-randomized controlled trials (a minority of the selected studies), cohort studies, cross-sectional studies, retrospective case studies, pilot interventions, and community-based intervention studies, although the designs were heterogeneous and inconsistently reported across all manuscripts.

The number of participants in the selected manuscripts that reported this number ranged from 17 to 154,934 participants per study. Yet, not all articles reflect the total number of enrolled participants: in some reports, these participants were divided over different phases of the project, whereas in other manuscripts that analyze more than one study, the participants are divided between those studies.

Of note, there were two cases of overlap between the included manuscripts. Greiner and colleagues (37) analyzed multiple research projects, including a study described in a dedicated separate manuscript first published by Friedman and colleagues (36). To avoid duplication in our analysis, we include Greiner’s study only when it focused on the remaining four studies not accounted for in the main study and refer to Friedman and colleagues’ study otherwise. Moreover, Sheppard and colleagues’ report (27) described complementary details about recruitment strategies and results to those included in Kreling and colleagues’ (28) manuscript. We include both manuscripts in this case: Kreling and colleagues (28) account for and measure the effectiveness of the broader research infrastructure they have put in place to serve the Latinx population in their catchment area. At the same time, Friedman’s work provides a narrower descriptive report of recruitment methods utilized in the research coalition (36).

In the following pages, we first narratively describe the barriers to participation identified in the retrieved articles. Second, outline the ways in which community members were involved in the design, implementation and dissemination of studies. Third, we unpack the recruitment practices evaluated as part of research teams’ efforts to improve participation among underserved groups. Noting that several studies combined more than one approach, we present those approaches in an individualized manner, following the themes developed throughout the narrative analysis. Each of them represent strategies to improve participation of minoritized groups in ED research: (1) rethinking recruitment locations and partnerships with local communities; (2) designing educational interventions and including peer-educators; (3) combining research with community needs; (4) increasing cultural competence of research teams; and (5) overcoming practical barriers in study design.

### 3.2 Barriers to cancer ED research participation

Each included study identified barriers to participation for underrepresented groups based on original research or existing literature. These identified barriers informed the rationale behind the intervention to increase research participation. Table 4 shows the frequency and percentages of the various obstacles identified in the sampled studies. Percentages represent the proportion of included studies (*N* = 38) that identified each barrier. Individual studies could report multiple barriers; therefore, percentages do not add up to 100%.

**Table 4:** Barriers to participation identified across included studies (*N* = 38).

Barrier identified	Studies reporting barrier, n	% of included studies (*N* = 38)
Mistrust	21	55%
Lack of knowledge	14	37%
Lack of trial awareness	12	32%
Geographic accessibility	8	21%
Lack of cultural competency in research	7	18%
Financial burden	4	11%
Time burden	4	11%
Fear of participation procedures	4	11%
Concerns about confidentiality/privacy	4	11%
Stigma/misconceptions about participation	4	11%
Group not approached for research	3	8%
Practical barriers (general)	3	8%
Uninsured/underinsured	2	5%
Fear of uncovering health problems	2	5%
Discrimination	2	5%
Language barrier	2	5%
Limited access to health services	2	5%
Comorbidities	1	3%
Limited access to public spaces	1	3%

Mistrust, the most cited barrier to cancer ED research participation among underrepresented groups, could refer to distrust of both healthcare and biomedical research (26, 31–33, 35, 36, 41, 48, 49, 53, 60, 62). Mistrust in healthcare and biomedical research can stem from historical wrongdoings, such as the infamous and widely cited Tuskegee Syphilis Study (63), as well as ongoing experiences of exclusion and discrimination in healthcare settings (53). Mistrust was coded separately from concerns about confidentiality, such as data breaches or information being passed on (31, 45, 61, 62), or privacy issues (31, 61), and fear of the clinical procedures one might undergo as part of cancer ED research, such as venipuncture or x-rays (30, 31, 42, 51, 61).

Perceived lack of knowledge of cancer, cancer early detection, and cancer ED research was also a commonly cited reason for nonparticipation (28–30, 32, 36, 37, 40, 43, 48, 51, 56, 57, 59, 60), as well as a lack of awareness that there are cancer ED trials that healthy people can sign up for (29, 30, 32, 33, 36, 40, 41, 43, 45, 50, 54, 59). While these barriers were often framed as a problem within the underrepresented community, we know that they can also be interpreted as a failure by research institutions to reach these groups, if not a failure of wider society to teach everyone basic knowledge about cancer and cancer research (29, 64)—a much smaller number of studies (34, 37, 45, 49, 55), placing the problem on the institutional side of the equation.

### 3.3 The role and extent to which community members were involved in research design, implementation and dissemination

Community-based participatory research approaches, meaning some participation and/or involvement of target communities in the intervention design, were common across the literature (1, 3, 5, 11, 13–15, 20, 21, 25, 27, 29, 33). These approaches took many forms, ranging from remunerated community advisory boards that oversaw research development (15), or involving community-based organizations in implementing the study (25)—at times combining multiple community-based participatory activities in one study. Table 5 summarizes the range of roles community members took on in the reviewed studies.

**Table 5:** Community roles in research design and implementation across included studies.

Community role	Description	Engagement practices
No involvement	Community not involved in research design or implementation.	Community engagement is not reported in the manuscript
Setting / access point	Community organizations provide venues or access to participants.	Churches, barbershops, community centers used for recruitment, education, screening, or data collection.
Research advisor	Community members provide input to inform research materials, messaging, or dissemination.	CAB review of materials, community feedback to support tailoring of recruitment messages and dissemination planning.
Research implementation partner	Community members actively support or conduct research activities.	Training of community ambassadors or educators to promote research and recruitment, co-development of recruitment materials through community testimonials
Research leader	Community members help define priorities and guide research decisions.	Community-initiated projects, CAB-driven research agenda, shared decision-making with researchers.

The meaningfulness of different community engagement or participatory approaches is an empirical question that was not addressed in the current study. This is because some manuscripts described their methods in too little detail to assess whether participation was substantive or merely nominal. Moreover, rather than a blueprint for research, or a collection of prescriptive practices, community-based participatory approaches can be considered as a series of values that researchers work toward, such as equitable power distribution, trust-building, and mutual commitment (65). Thus, the 13 publications this study identified as following community-based participatory approaches because their authors claimed to work toward those principles in some capacity, including some or all outlined involvement and engagement practices presented on the table.

### 3.4 Approaches to improve participation of minoritized groups in cancer ED research

**Figure 2:**
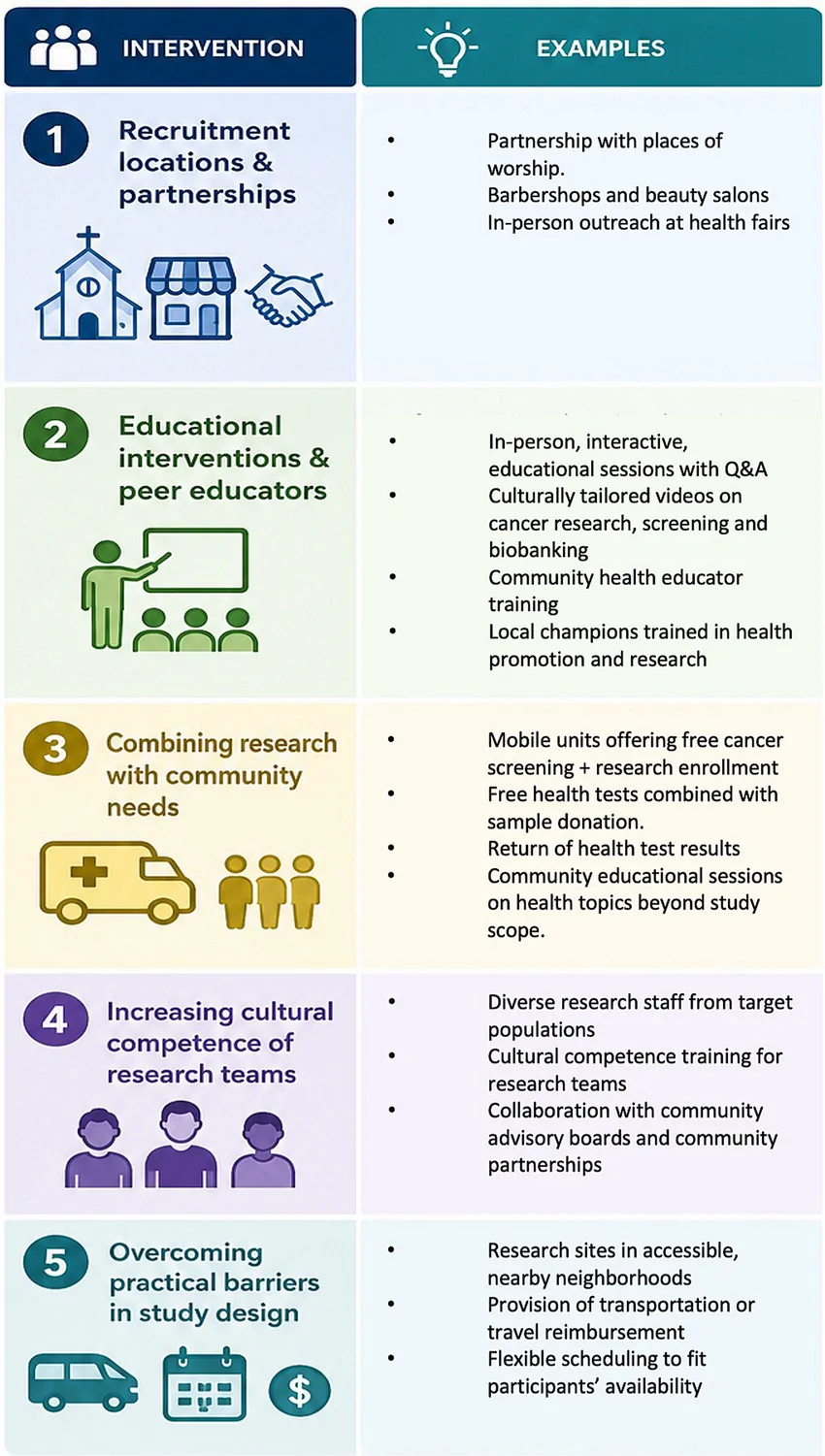
Approaches to improve participation of minoritized groups.

The included papers displayed a high level of heterogeneity and often combined different recruitment and participatory strategies. Therefore, the following highlighted strategies should not be interpreted as distinct, singular solutions but rather as tools that can be employed in various combinations to improve the participation of underserved groups in cancer ED research. Figure 2 presents a visual summary of the main strategies and examples from the literature that demonstrated increased participation of minoritized groups in.

#### 3.4.1 Recruitment locations and partnerships

With mistrust in clinical research and healthcare being one of the critical barriers to research participation, solutions for recruiting and involving people from minoritized groups in cancer ED research often focused on engaging potential participants in settings that inspire trust. This included spaces with trusted ‘gatekeepers’: places of worship, healthcare centers, and barbershops. To approach people there for research participation, it thus used ‘trust by proxy’, where the environment and people associated with it facilitate trust-building between research teams and potential participants.

##### 3.4.1.1 Church

A remarkably high volume of studies predominantly chosen to reach Black participants focused on establishing partnerships with places of worship: churches (28, 30, 31, 33, 35, 36, 38, 44, 46, 48, 61) or, in one case, temples (58). Churches frequented by a particular minoritized group were approached by researchers and asked to either allow recruitment via the church’s communication channels or to set up a more comprehensive (community-based) programs combining long-term research with other activities. A successful example is the study by McNeill and colleagues (44), who developed a partnership with three African American churches in Houston, one of them with as many as 15,000 members, for a large cohort study. The research team spent 1 year building a trusting relationship with the churches. In addition to these dialogs, research teams attended worship programs, responded to requests for talks on cancer control, and guided church members to local screening services. The trusting, reciprocal relationship between the research team and the churches led to an overwhelmingly positive response to the recruitment efforts. Similarly, Slade and colleagues (48) developed a trusting relationship with the leadership of participating churches, who then helped researchers plan research that limited barriers to participation, for example, by scheduling events at the church around activities that would already draw people to church.

##### 3.4.1.2 Barbershops and salons

Five studies used barbershops and beauty salons to reach potential participants from underserved groups for cancer ED research participation (33, 37, 41, 46, 47). Since barbershops and salons are often gender-segregated, they facilitate reaching people from a particular gender and provide an environment where a (sex-specific) cancer can be discussed without the presence of the other sex. In addition, Jones, Steeves, and Williams (41) note that barbershops have historically been one of the few places where ‘African American men, regardless of their education, socioeconomic status, or occupation, could congregate and voice their opinions about various topics’. Therefore, the barbershop is a location where conversations about cancer, which will affect many men in their lifetime, and research participation can be introduced.

##### 3.4.1.3 Healthcare centers

Ten studies chose clinical settings as one of the recruitment sites (28, 34, 37, 39, 42, 43, 50, 53, 54, 58). Recruitment strategies included could be a letter or message from a physician (50, 58), multiple callbacks from the clinic (34), or face-to-face recruitment at either a community-based or specialist clinic (12, 14, 17, 32, 34, 35, 37). Tabriz and colleagues (50) attempted to recruit participants through an online patient portal, which would mitigate human bias in trial invitations. Still, they found that this recruitment method led to less ethnically diverse study samples as Black and Asian primary care patients were less likely than White patients to read the portal message, opening the attached link in the message, and consenting to participate in the trial. Similarly, in a study by Wang and colleagues (29), a bilingual, signed physician’s letter sent to Chinese Americans saw an enrolment rate of 32% on average, but this enrolment strategy enrolled comparatively fewer recent immigrants and uninsured Chinese Americans than community-based approaches. These findings illustrate that this recruitment approach risks excluding groups with less trust in healthcare institutions.

#### 3.4.2 Designing educational interventions and including peer-educators

Since lack of knowledge about cancer ED and research was identified as a significant barrier to research participation, interventions promoting education and awareness were often included as part of recruitment strategies. Fourteen studies organized educational sessions to counter stigma, stereotypes and false information about cancer research and associated data-collection procedures (10, 28, 30, 31, 36, 40, 42, 43, 45, 49, 51, 55, 56, 59), hypothesizing that increasing the knowledge about cancer, early detection, and cancer ED research in communities would lead to higher participation rates. Smith and colleagues (49) found that educational workshops followed by question-and-answer sessions were the most successful recruitment strategy, the latter being essential to give participants space to get their questions answered instead of being passive recipients of information. Another practical approach among these studies was to ask those present at the educational session to participate in the data collection immediately afterwards. However, this type of intervention was only feasible when participants were asked to either sign up to study, donate a biospecimen sample, or complete another brief test or task (10, 30, 42, 45, 56). Four studies designed interventions through culturally tailored videos to raise awareness of biobanking and cancer screening research projects (26, 32, 56, 61). Hainsworth and colleagues, for example, brought together a group of African American men to inform the development of a video to raise awareness of prostate cancer and promote their participation in cancer research. The group recommended leading with a positive case study and including family members as a target audience, as they could encourage their loved ones to participate in cancer screening. They also recommended targeting younger African American men, who may likewise be affected. The group also advised for the use of lay rather than scientific language, statistics, or portrayals of researchers (26).

##### 3.4.2.1 Peer educators

Seven papers from the selected studies recruited and trained peer educators from the target community to disseminate information about cancer (ED) and research and recruit potential study participants. Peer educators, described by various terms such as ‘*Promotores de Salud*’ (29, 55), ‘community health educators’ (32, 59), or ‘local champions’ (62) were chosen to disseminate information in a particular community because they are more likely to be considered a trusted source than a research team of ‘outsiders’, and because they are more likely to understand cultural sensitivities and appropriate manners of communication. Therefore, recruiting peer educators not only addressed knowledge gaps in cancer, early detection, and research but also addressed the lack of trust in biomedical research and healthcare professionals, as well as the importance of cultural appropriateness. In addition, peer educators can reach community members through their existing networks, thereby reaching more isolated individuals (55).

Involving peer educators in study design and implementation also often seemed coupled with a desire to alter power dynamics between research teams and communities involved in cancer ED research. In contrast with one-off recruitment strategies, peer educators can be part of a long-term relationship between research teams and their communities and become essential actors in the bidirectional communication between the two sides. Moreover, training peer educators (e.g., in presentation skills, leadership skills, health promotion and recruitment strategies) and investing in their development can be part of an effort to ‘give back’ to the researched community and to make the relationship between researchers and communities mutually beneficial. The *promotoras* participating in the study by Larkey and colleagues (55) developed skills and obtained experiences that led to further opportunities for career advancement.

#### 3.4.3 Combining research with community needs

People approached for cancer ED research may not see cancer ED research as a priority among all other causes and activities that require their time and attention. Moreover, participation in research activities can be an unappealing prospect. Therefore, seven studies in the sample combined research activities with community needs to make participation more appealing and to provide essential information to communities that could benefit from this. As a result, incorporating services that address community needs can rendered the researcher-participant relationship less extractive and contributed to a trusting relationship between the research team and research participants from marginalized communities.

##### 3.4.3.1 Screening service and research participation

Three studies invited research participants by bringing screening services into the communities researchers hoped to recruit from (10, 30, 54). For example, McElfish and colleagues (54) recruited rural, medically underserved women in Arkansas (USA) for their breast cancer study using a mobile mammography unit (MammoVan). The unit provided breast cancer screening to women without a history of breast cancer free of charge, after which the women were asked to participate in the study, which involved an additional survey and saliva sample. The MammoVan recruitment strategy led to more rural and lower-educated participants (54). In the other two studies, potential research participants were offered several tests to collect health data, after which they were asked to donate these samples for research purposes (10, 30).

This approach successfully reached underrepresented groups in cancer ED research in all three studies, but two things must be noted. Firstly, this method only has added value in areas where access to screening and other health services is insufficient: in places where potential research participants have easy access to cancer screening services and other health tests, this approach would have little added value for communities that researchers hope to recruit from. Secondly, the types of procedures offered to and asked from potential participants influence the likelihood of participation: Yeary and colleagues found that research activities with a higher participant burden, such as mammography and blood tests, saw much lower participation rates than simple procedures, such as surveys and anthropometric measurements, even when the indicated intent to participate was equally high or higher (10).

##### 3.4.3.2 Educational needs and research participation

Rather than offering insight into personal health, four studies (43, 44, 48, 52) combined research recruitment with meeting the educational needs of a particular group or community. Educating about health topics was shown to increase intent to participate in research (61), but offering educational sessions about various issues can also be part of building trust between researchers and participants and an act of service to a participating community. This is illustrated by a study conducted by Langford and colleagues (43), for which the research team organized breakfasts for African American men. In addition to talks about prostate cancer and cancer research, the team solicited ideas from men to address topics they wanted to learn about. As a result, some breakfast talks covered seemingly unrelated themes, such as self-motivation and estate planning, demonstrating the researchers’ recognition of the broad way in which men define health. By inviting various speakers, from NFL players to urologists, to the breakfast program, the research team offered their resources to benefit the community.

#### 3.4.4 Increasing cultural competence of research team

The cultural competence of research teams and outreach materials was an overarching and often-cited theme in the selected studies. However, in six studies (27, 28, 31, 44, 52, 58), increasing the cultural competence, or cultural humility (66) of the research and recruitment staff, was a key strategy. In all cases, manuscripts mentioned having a diverse team that included members of the community the study sought to engage. In addition, some research teams were explicitly trained in cultural competence. For example, Wallington and colleagues (52) employed a highly diverse research and recruitment team, who were all trained in core practices of cultural competence, “acquiring and institutionalizing cultural knowledge” (52). While the effects of diverse research teams and cultural competence training are challenging to quantify, results seemed optimistic: Wallington and colleagues reported that the culturally competent, community-based strategies led to a 62% increase in the enrolment of black participants in the study compared to previous recruitment tactics (52).

#### 3.4.5 Overcoming practical barriers in study design

Sixteen studies mentioned practical barriers to research participation for potential participants (10, 26, 28, 35, 36, 41, 44, 46, 50, 52, 54, 55, 58–60, 62, 67). These barriers include geographic accessibility, that is, the location where research was being conducted were far or difficult to reach (36, 41, 46, 52, 54, 60), or participants had no access to the right transportation (35, 41, 55, 60); and the time, duration, or timing of trials (33, 55, 60, 62), language barriers (58, 59), among other constraints. These practical barriers can be felt disproportionally by ethnic minority populations because they often have a lower socioeconomic status compared to their White counterparts (68).

For issues related to the geographic accessibility of the research location, different solutions were offered, which focused either on bringing the research site closer to potential participants or facilitating transportation. For example, Wallington and colleagues (38) moved the site from where research and outreach were being conducted to two community-based offices in the medically underserved neighborhoods where they hoped to recruit for the study. The locations were picked with input from two community advisory boards and were both well-connected to bus and metro lines, as well as being within walking distance for many study participants. To facilitate transportation to the research site, a solution offered by studies was to pay for or reimburse transportation expenses (26, 32, 56, 61) while colleagues organized transportation to the churches that functioned as project sites in their most successful recruitment arm (35).

## 4 Discussion

This study has highlighted several ways to include groups often underrepresented in cancer ED research. It draws on a scoping review of studies published between 2002 and April 2022 in anglophone countries – primarily the USA, that have empirically assessed recruitment strategies and interventions to enhance participant diversity. We have presented the identified strategies around five core principles used to group them: (1) rethinking recruitment locations and partnerships with local communities; (2) designing educational interventions and including peer-educators; (3) combining research with community needs; (4) increasing cultural competence of research teams; and (5) overcoming practical barriers in study design.

Our study contributes to the literature by providing evidence of the relevance of strategies include those that foster long term partnerships with communities by cultivating reciprocity and listening to community needs, honoring those needs within the resources, limits, and abilities that research teams have. We also show evidence of successful strategies focused on promoting understanding through educational interventions that are culturally aligned and have input from the communities, so that agenda-setting becomes a collaborative project. These interventions not only helped raise awareness of cancer, the importance of early detection, and the availability of clinical studies; they also sought to intervene on the power asymmetries between research and minoritized communities through reciprocity, offering information and resources for the professional advancement of members of the community themselves (28, 44, 47). This study also demonstrates that in-person interactions, whether by the research team or designated (peer) recruiters, are important in engaging participants from minoritized groups (35, 46, 67). The importance of face-to-face interactions supports building trust in the community and enhance the research team trustworthiness: practices include getting to know the research team in person and having a two-way dialog to address particular reservations of potential research participants (69, 70). Gren and colleagues (38) found that for the recruitment of minoritized populations, in-person ‘community outreach’ (e.g., recruitment in churches, seminars, and health fairs) was an important addition to direct mail communication, as direct mail alone failed to convince people from minoritized groups to participate in research. Existing literature suggests several locations where such face-to-face interactions could occur, as well as forms of interaction (such as educational workshops and mobile screening services) and aspects to consider when communicating with potential research participants (e.g., culturally tailored-messages including testimonials from ethnically- matched participants or researchers can promote resonance of the message within the community).

To promote cultural alignment that does not essentialize ethnic communities and recognizes the internal heterogeneity within populations, studies demonstrate the importance of developing study protocols and recruitment strategies in collaboration with people from the communities researchers hope to recruit from. The engagement strategies identified were virtually all developed with at least some input from participants or community advisors who belong to an underrepresented group. For example, Cunningham-Erves and colleagues report on a study to explore trust and distrust among Black communities in Tennessee. The whole initiative was initiated by the Case Comprehensive Cancer Center’s community advisory board (32). Rogers and colleagues asked a community advisory board in Utah to give feedback on the content and presentation of recruitment materials (46). Davis and colleagues worked with their community advisory board to develop their study recruitment plan, infusing it with practical perspectives to reach subpopulations within a diverse population of Blacks in Tampa Bay in the USA (33). Slade and colleagues worked with a community advisory board across 2 church-based outreach initiatives. Advisory panel members provided feedback about the strategies the team proposed to use to engage the churches and the cultural appropriateness of the proposed content included in educational materials and engagement procedures. The advisory panels also offered guidance and assisted in identifying churches for engagement and recruitment to serve as potential participants in the interventions (48). Thus, 13 studies chose community-based participatory approaches and developed protocols and/or outreach strategies through collaboration or co-creation with individuals or organizations representing minoritized groups (10, 26, 29, 31, 33, 37, 44, 46, 48, 49, 52, 53, 59). These approaches communicate respect for and attention to minoritized groups researchers want to engage in cancer ED research; they have also been shown to increase statistically positive outcomes in a recent systematic review (23).

This study also demonstrates that combinations of strategies for involving participants from underrepresented groups are necessary. Each recruitment and engagement strategy successfully reaches some groups but excludes others. For example, Sadler and colleagues (47) note that the Black women they recruited through the cosmetologist’s salons are women who can afford such services. Similarly, Leach, Schoenberg, and Hatcher (60) found that, though they conducted research in a rural area characterized by low education levels and high breast cancer mortality, the women they got in touch with through outreach efforts tended to enjoy a higher socioeconomic status and were up to date on breast cancer screening. In other words, even when successful overall, strategies to invite underrepresented groups to participate in cancer ED research tend to exclude specific marginalized subgroups. This suggests that various engagement and recruitment strategies should be combined to reach a wide range of potential participants, and researchers should pay careful attention to their study designs to address the concerns of these (sub)groups. Again, community-based-participatory approaches can help research teams identify these barriers accurately.

This scoping review documents a plethora of barriers and issues in cancer ED research and biomedical research more generally, experienced particularly by members of minoritized groups. These include a lack of trust in researchers, a limited of medical literacy, and insufficient cultural competence in research staff. Although we offer tools to address some of these issues and barriers within a single study or project scope, addressing these issues requires a long-term, collective effort from research institutions and healthcare organizations more generally. Together, these efforts contribute to a more significant shift in biomedical research culture that (re)builds trust between researchers and potential research participants, especially those from minoritized backgrounds. To build trust, relationships between researchers and research participants need to change from appearing extractive to feeling mutually beneficial and collaborative (8). The engagement of minoritized groups in research and inclusive study designs are just one part of this process; increased education and career opportunities in cancer research for people from minoritized backgrounds (71, 72), input from minoritized groups at the policy level, and improved ethnicity coding in healthcare (73) are other essential elements.

This study has limitations. The searches in PubMed and PsycINFO were executed exclusively in English. While many of the research studies discussed have assessed interventions that could be applied in a variety of contexts and countries, they are based on healthcare systems, socio-economic conditions, and racialized societal relations that may be unique to their own countries, which in turn affect the perceived barriers to cancer ED research. In addition, the search terms used in this study leave out studies that do not mention recruitment strategies in their title or abstract. Therefore, we may have missed publications for which recruitment strategies are not a focus of the publication, but which are nonetheless described and evaluated in the body of the text. The meaningfulness of different community engagement or participatory approaches is an empirical question that was not address in the current study. This because some manuscripts described their methods in too little detail to assess whether participation was substantive or merely nominal. Finally, this study only includes manuscripts that have assessed or qualitatively evaluated interventions to increase participation or engagement of underserved groups, which means the timeline of these studies is limited: projects that aim to address the historical mistrust of some groups toward cancer research and foster a culture of trust between researchers and research subjects are more challenging to evaluate since they take much longer to bear fruit (74).

Future studies may be necessary to assess the effectiveness of specific interventions in engaging underrepresented groups compared to ‘traditional’ or known recruitment and engagement strategies. Importantly, no intervention will work for all, so evaluations will have to pay careful attention to the particularities of, and heterogeneity within, each underrepresented group (7) and the context in which they live. In addition, future studies are needed to systematically assess the potential for contemporary communications methods to recruit participants from underrepresented groups into cancer ED research. Some approaches using direct mail and radio commercials included in the review may become irrelevant in many societies today, whereas various social media platforms have shown some promise to reach potential cancer ED research participants (75).

Lastly, it is important to note that most of the manuscripts identified in this study report barriers and interventions to promote participation in research among Black and Hispanic/Latinx groups, whereas other (ethnic) minorities received less attention (i.e.: Asian, rural communities), or were absent (i.e.: sexual minorities and people with disabilities and people with co-morbidities). Devising and assessing strategies to improve the recruitment and engagement of these other groups is urgently needed. These efforts will continue enhancing the diversity of participant research cohorts and contribute to a general shift in research culture. The shift was bearing fruit, as evidence of national initiatives such as the National Cancer Institute initiative in the USA to promote research informed by communities’ local cancer needs, or the still ongoing investment by Cancer Research UK funding programs to embed community engagement approaches and attention to inequity across their research portfolio.

## 5 Conclusion

This scoping literature review has highlighted empirically tested tools that research teams can employ to improve participation rates of groups often underrepresented in cancer ED research. Combinations of these methods can help overcome the perceived barriers to participation in cancer research that mainly affect people without a cancer diagnosis from these minoritized groups. Not only will these methods lead to more representative cancer ED studies that benefit all of society; the highlighted approaches also contribute to a more significant shift in research culture toward less extractive and more trusting relationships between researchers and the public.
