# Supplementary material for: Increasing participation of underrepresented groups in cancer early detection research: a scoping review
Source: Front Public Health. 2026 Aug 19;14:1841107. doi: 10.3389/fpubh.2026.1841107 (PMC13537035; doi:10.3389/fpubh.2026.1841107)
Supplement: Supplementary file 2 [file Supplementary_File_2.docx]

**Systematic Literature Review – Second Reviewer Instructions**

On Pubmed, copy and paste these strings in the search box and search them separately:

1: ("clinical trial"[Title/Abstract]) OR ("early detection research"[Title/Abstract]) OR ("cancer research"[Title/Abstract])

2: (underrepresent*[Title/Abstract]) OR (minorit* [Title/Abstract]) OR (underserved[Title/Abstract]) OR (hard-to-reach[Title/Abstract]) OR (marginal*[Title/Abstract]) OR (vulnerable[Title/Abstract]) OR (disadvantaged[Title/Abstract]) OR (racial*[Title/Abstract]) OR (ethnic*[Title/Abstract]) OR (BIPOC[Title/Abstract]) OR (BAME[Title/Abstract]) OR ("South Asian"[Title/Abstract]) OR (Asian[Title/Abstract]) OR ("African American"[Title/Abstract]) OR (Latin*[Title/Abstract]) OR (Black[Title/Abstract]) OR (Hispanic[Title/Abstract]) OR ("South American"[Title/Abstract]) OR (Indigenous[Title/Abstract]) OR ("Native American"[Title/Abstract]) OR (Aboriginal[Title/Abstract]) OR (migrant[Title/Abstract]) OR (immigrant[Title/Abstract]) OR (disabil*[Title/Abstract]) OR (disabl*[Title/Abstract]) OR (neurodiverse[Title/Abstract]) OR (handicap*[Title/Abstract]) OR (sexual minorit*[Title/Abstract]) OR (gender minorit*[Title/Abstract]) OR (LGBT*[Title/Abstract]) OR (gay[Title/Abstract]) OR (lesbian[Title/Abstract]) OR (rural[Title/Abstract]) OR ("socio-economic status"[Title/Abstract]) OR (working-class[Title/Abstract])

3: (recruit*[Title/Abstract]) OR (participa*[Title/Abstract]) OR (engagement[Title/Abstract]) OR (retention[Title/Abstract]) OR (involvement[Title/Abstract]) OR (enrolment[Title/Abstract]) OR (enrollment[Title/Abstract]) OR (accrual [Title/Abstract])

4: (cancer[Title/Abstract]) OR (oncol*[Title/Abstract]) OR (lymphoma[Title/Abstract]) OR (carcinoma[Title/Abstract]) OR (adenoma[Title/Abstract]) OR (melanoma[Title/Abstract]) OR (myeloma[Title/Abstract]) OR (sarcoma[Title/Abstract]) OR (leukemia[Title/Abstract]) OR (carcinoid[Title/Abstract]) OR (tumor[Title/Abstract]) OR (astrocytoma[Title/Abstract])

Then, after having searched all four, click on ‘advanced’ below the search box. At the bottom of this page, there now should be a history of your past few searches. It should look like this (ignore the fact that my searches start at #2, I had to delete a search, so pretend it goes #1 to #4).

Then, type or copy-paste into the query box: #1 AND #2 AND #3 AND #4

(This should give about 1,137 results)

Now, adjust the time bar so that only papers from 2002 onwards are shown.

I’d recommend adjusting the page settings so that it displays 200 articles per page. It is automatically sorted by best match.

You can now scan the titles of the first ~235 (20%) – most will clearly not fall within the criteria, but some may require reading the abstract or even scanning the article.

The inclusion/exclusion criteria are as follows:

| **INCLUSION CRITERIA** | **EXCLUSION CRITERIA** |
| --- | --- |
| Research undertaken in High-Income Country (e.g. USA, UK, Western Europe, Canada, Australia, New Zealand, Hong Kong) | Research undertaken in Low- or Middle-Income country |
| Participants aged 18 years or above | Participants under 18 years old |
| Published in or after 2002 | Published before 2002 |
| Peer reviewed publication | Not peer reviewed and/or not yet published |
| Paper relates directly to cancer - all cancers, gene mutations associated with cancer (e.g. BRCA1/BRCA2), viruses associated with cancer (e.g. HPV) | Paper does not relate to cancer |
| Purpose of study is not therapeutic/service but cancer research* | Research is therapeutic – participants participate to better their own health outcomes* |
| Research involves participants that have not been diagnosed with cancer (pre-diagnostic) | Research participants are diagnosed cancer patients or cancer survivors |
| Paper focuses on recruitment, participation, or engagement of underserved groups | Paper focuses on recruitment, participation or engagement of general public, not specific underserved groups |
| Intervention to improve recruitment, participation or engagement is implemented/tested | Intervention to improve recruitment, participation, or engagement is only suggested, not implemented/tested |
| Implemented intervention is evaluated, quantitatively or qualitatively | Implemented intervention is only described, outcomes or implementation is not quantitatively or qualitatively evaluated (e.g. protocol) |

*This is difficult to define, but the intent of the study is key: is it research, or providing a service? If the research produces secondary incentives for participants (e.g. receiving results of mammogram) that is allowed, but if the purpose is therapeutic (e.g. cancer patients have the chance to receive novel treatments) this is excluded.
